# Fetal dextro-transposition of the great arteries: a narrative review of management and neurodevelopmental outcomes

**DOI:** 10.3389/fphar.2026.1926005

**Published:** 2026-09-11

**Authors:** Yang Zhang, Zubo Wu

**Affiliations:** 1 Department of Obstetrics and Gynecology, Union Hospital, Tongji Medical College, Huazhong University of Science and Technology, Wuhan, China; 2 Department of Pediatrics, Union Hospital, Tongji Medical College, Huazhong University of Science and Technology, Wuhan, China

**Keywords:** artificial intelligence, fetal complete transposition of the great arteries, neurodevelopment, perinatal management, prenatal diagnosis

## Abstract

Fetal dextro-transposition of the great arteries (d-TGA) represents a severe cyanotic congenital heart defect. Without effective intracardiac communication, affected neonates face life-threatening hypoxemia and acidosis shortly after birth. Recent investigations have unveiled an additional dimension: d-TGA patients experience compromised cerebral development and inadequate oxygen delivery beginning *in utero*, subsequently manifesting as executive dysfunction and other neurodevelopmental challenges throughout childhood and adolescence—obstacles that now constitute the primary barriers to favorable long-term outcomes. Advances in prenatal ultrasonographic diagnosis, artificial intelligence-assisted technologies, and integrated multidisciplinary perinatal management have substantially elevated survival rates in recent years. Nevertheless, persistent clinical challenges remain: suboptimal prenatal detection rates, incomplete genetic counseling frameworks, insufficiently delineated perinatal treatment pathways, and delayed interventions for neurodevelopmental impairments. This narrative review integrates current knowledge regarding fetal d-TGA, endeavoring to provide actionable insights for clinical diagnosis, therapeutic strategies, and comprehensive management.

## 1 Introduction

Dextro-transposition of the great arteries (d-TGA) in the fetus arises from aberrant rotation and resorption of the embryonic conus during early cardiac morphogenesis, culminating in ventriculoarterial discordance with atrioventricular concordance—a complex congenital cardiac malformation. Its cardinal anatomical hallmark involves the aorta arising from the right ventricle while the pulmonary artery originates from the left ventricle, thereby establishing complete separation between systemic and pulmonary circulations. This entity must be clearly distinguished from congenitally corrected transposition of the great arteries (cc-TGA), in which both atrioventricular and ventriculoarterial discordance coexist (“double discordance”), resulting in a distinct pathophysiology, natural history, and management pathway (Martins and Castela, 2008). Given that the overwhelming majority of d-TGA cases exhibit normal atrial situs with the aorta positioned anteriorly and rightward to the pulmonary artery, this arrangement is designated dextro- (d-) transposition to differentiate it from levo- (l-) or congenitally corrected forms. This entity accounts for 5%–7% of all congenital heart diseases, presenting with a prevalence of approximately 0.02%–0.04% among live births and demonstrating a male predominance with a ratio of 2–3:1 (Vigneswaran et al., 2017). Classification hinges upon the presence of associated cardiac anomalies, delineating two principal categories: (1) Simple d-TGA: characterized by the absence of ventricular septal defect (VSD) or the presence of hemodynamically inconsequential VSD (<1/3 of the aortic valve annulus diameter), representing approximately 50%–70% of cases; (2) Complex d-TGA: distinguished by one or multiple coexisting cardiac malformations, among which VSD predominates, while additional anomalies encompass left or right ventricular outflow tract obstruction, aortic arch lesions, atrioventricular valve pathology (stenosis, atresia) accompanied by unilateral ventricular hypoplasia, and systemic venous return abnormalities (Villafañe et al., 2014). These anatomical distinctions profoundly influence postnatal hemodynamics. Simple d-TGA presents particularly formidable challenges: absent patent foramen ovale, VSD, or patent ductus arteriosus—conduits essential for blood mixing—affected infants cannot establish viable oxygenated circulation, precipitating potentially fatal hypoxemia and acidosis (Warnes, 2006). Furthermore, emerging evidence illuminates that d-TGA patients experience aberrant cerebral development and compromised oxygen delivery commencing *in utero*, subsequently manifesting as executive dysfunction and allied neurodevelopmental impairments throughout childhood and adolescence—complications that have crystallized as principal obstacles in long-term follow-up and outcome optimization.

Current clinical management of fetal d-TGA confronts several pressing imperatives: inadequate prenatal detection rates, incomplete genetic counseling frameworks, insufficiently standardized perinatal treatment algorithms, and suboptimal early identification coupled with intervention for neurodevelopmental impairments. Concurrent advances in prenatal ultrasonographic diagnosis, AI-assisted diagnostic platforms, and integrated multidisciplinary perinatal care paradigms have markedly elevated survival rates. Clinical management priorities for fetal d-TGA are shifting from survival alone toward long-term quality of life, with early recognition and intervention for neurodevelopmental impairment now recognized as a critical determinant of long-term outcomes. As a narrative mini-review, this article synthesizes key primary studies, guideline documents, and recent meta-analyses identified through targeted searches of PubMed/MEDLINE, prioritizing publications from the past decade and foundational earlier work. Therefore, this review examines recent advances in fetal d-TGA from both mechanistic and clinical perspectives, while projecting future research trajectories.

## 2 Pathogenesis of fetal d-TGA

Between gestational weeks 5–8, the conotruncus undergoes approximately 180° of spiral rotation, positioning the aorta rightward and posterior to the pulmonary artery. During normal cardiac ontogeny, the subpulmonary conus persists as muscular tissue while the subaortic conus undergoes involution; conversely, in fetal d-TGA, excessive development and anterior displacement of the subaortic conus coupled with relative atrophy of the subpulmonary conus generates abnormal spatial relationships between the great arteries (Bartelings and Gittenberger-de Groot, 1991).

The etiology of d-TGA involves intricate interplay between genetic and environmental determinants. From a genetic standpoint, approximately 5%–10% of fetal d-TGA cases harbor chromosomal aberrations, comprising aneuploidies (2%–3%), 22q11.2 microdeletion syndrome (1%–2%), and other chromosomal microdeletions/duplications (2%–5%) (Blue et al., 2017). Monogenic mutations are detected in roughly 5%–15% of sporadic cases, with literature documenting associations between d-TGA and pathogenic variants in cardiac developmental genes, including those within the Nodal signaling pathway (*CFC1*, *FOXH1*), as well as gene *ZIC3*, *MED13L*, and *WNT5A* (Blue et al., 2017).

Regarding environmental factors, the hyperglycemic milieu of gestational diabetes may augment fetal d-TGA risk through mechanisms involving oxidative stress induction, disruption of cardiac neural crest cell migration, and perturbation of epigenetic regulation (Blue et al., 2017). Huida and colleagues conducted a prospective cohort investigation revealing significant associations between maternal first-trimester obesity (BMI ≥30 kg/m^2^) and offspring d-TGA risk, while elevated C-reactive protein levels and leukocyte counts during early pregnancy suggested that maternal low-grade inflammatory states might contribute to pathogenesis (Huida et al., 2023). Additionally, first-trimester exposures to alcohol, ozone, arsenic, certain medications (including retinoic acid and antiepileptic agents), or rubella virus infection constitute recognized risk factors for fetal d-TGA development (Paparella et al., 2025; Wang et al., 2025; Suhl et al., 2023).

## 3 Prenatal diagnosis of fetal d-TGA

Fetal echocardiography serves as the cornerstone for prenatal d-TGA diagnosis, yet detection poses considerable challenges. Simple d-TGA demonstrates prenatal detection rates of merely 3%–27%, with overall detection rates below 50%, rendering it highly susceptible to oversight. When d-TGA escapes prenatal diagnosis, affected neonates frequently experience diagnostic delays and ductal closure, precipitating severe hypoxemia and acidosis that jeopardize survival. Conversely, timely prenatal diagnosis substantially reduces neonatal acidosis incidence (3% vs. 18%), facilitates planned neonatal resuscitation and early surgical intervention, and improves neurodevelopmental trajectories (Donofrio et al., 2013; Calderon et al., 2012; Selvanathan et al., 2024).

The internationally recognized systematic diagnostic approach employs segmental cardiac analysis, sequentially evaluating atrial situs, atrioventricular connections, and ventriculoarterial relationships (Escobar-Diaz et al., 2015). Strategies pivotal for enhancing d-TGA detection encompass: (1) Routine incorporation of outflow tract views: Evaluation cannot rely solely upon four-chamber views but must include left and right ventricular outflow tracts alongside three-vessel views; (2) Vascular course tracing: Ascending from ventricles to trace great arteries, confirming connections between the aortic arch and ductus arteriosus; (3) Color Doppler application: Demonstrating parallel great artery courses and the characteristic “double-ring sign”; (4) Focused screening in high-risk populations: For mothers with diabetes, first-trimester obesity, or prior offspring with congenital heart disease, dedicated fetal echocardiography is advisable (Yagel et al., 2001).

Fetal cardiac MRI, complementing echocardiography, offers superior visualization of great artery spatial relationships, aortic arch trajectories, and associated malformations, proving invaluable when ultrasonographic diagnosis proves challenging or inconclusive. While MRI elevates diagnostic accuracy for complex congenital heart disease by 15%–20% (Sarris et al., 2017), protracted examination duration, substantial costs, and requirements for relative fetal quiescence have precluded its adoption as a routine screening modality.

Artificial intelligence (AI) technology has progressed from theoretical frameworks into clinical validation phases across three distinct tasks that should not be conflated: (1) standard view classification. A 2024 systematic review encompassing 28 studies demonstrated that deep learning algorithms achieve 85%–92% accuracy specifically in automated identification and quality assessment of standard fetal cardiac views (e.g., four-chamber, outflow tract, three-vessel views), a task analogous to image quality control rather than diagnosis (Liu et al., 2024); (2) fetal cardiac abnormality detection. AI models incorporating convolutional neural networks have shown capability in flagging abnormal great artery spatial relationships and structural patterns such as the “double-ring sign” for general anomaly screening (Mayer et al., 2024); however, a 2025 meta-analysis found that AI performance data remain limited when distinguishing normal from abnormal fetal hearts across the full spectrum of malformations (D’Alberti et al., 2025); (3) lesion-specific diagnosis of d-TGA. direct evidence for AI accuracy in definitively diagnosing d-TGA as a specific lesion remains considerably more limited than the view-classification data above, and comparative work evaluating AI performance against the UK National Screening Program for hypoplastic left heart syndrome detection found that expert sonographer qualitative assessment still outperformed AI (Holt et al., 2025). Consequently, current evidence supports AI as an adjunctive tool for enhancing image quality and flagging potential anomalies for expert review, but does not yet support its use for autonomous lesion-specific diagnosis of d-TGA.

## 4 Genetic counseling for fetal d-TGA

For prenatally diagnosed d-TGA fetuses, following comprehensive informed consent from expectant mothers and families, prenatal diagnostic testing is recommended, including chromosomal karyotyping or chromosomal microarray analysis, with consideration of whole exome sequencing when indicated, to evaluate potential genetic aberrations.

d-TGA predominantly manifests as sporadic cases conforming to multifactorial inheritance patterns. For families having previously conceived a child with d-TGA, recurrence risk during subsequent pregnancies warrants stratification by disease subtype: Simple d-TGA: Sibling recurrence risk for d-TGA approximates 0.27%–0.82%, with risk for any congenital heart disease reaching 1.4%–2%; Complex d-TGA: Sibling recurrence risk escalates to 2%–3%, with risk for any congenital heart disease spanning 3%–5%; Parental d-TGA: Offspring recurrence risk approximates 3%–5% (Villafañe et al., 2014; Blue et al., 2017). While sibling recurrence risk exceeds that of the general population (background congenital heart disease risk: 0.8%, d-TGA prevalence: 0.02%), absolute values remain relatively modest. Factors modulating recurrence risk include: coexisting heterotaxy syndromes (recurrence risk reaching 10%–25%), identification of pathogenic monogenic mutations (recurrence risk 25%–50%), concurrent extracardiac malformations or developmental delays, and multiplex family history of congenital heart disease augmenting risk (Villafañe et al., 2014; Blue et al., 2017); conversely, isolated, simple d-TGA with negative family history and unremarkable genetic testing attenuate risk. Recurrence risk estimates above are derived primarily from general congenital heart disease cohorts and genetics-focused reviews (Gill et al., 2003), and d-TGA-specific subtype data remain relatively sparse; figures should therefore be interpreted as approximate ranges for counseling purposes rather than precise, universally validated values. Overall, among complex congenital heart diseases, d-TGA demonstrates relatively infrequent associations with defined genetic syndromes, with approximately 90% of cases representing isolated, non-syndromic sporadic occurrences typically lacking explicit family history, conferring relatively modest offspring recurrence risk (Villafañe et al., 2014; Blue et al., 2017), though this proportion may vary depending on the diagnostic modalities used (e.g., routine chromosomal microarray or exome sequencing versus karyotype alone) and the extent of extracardiac phenotyping performed.

## 5 Pregnancy and perinatal management of fetal d-TGA

Following prenatal d-TGA diagnosis, multidisciplinary teams encompassing obstetrics, fetal medicine, pediatric cardiology, genetics, and neonatology should collaboratively provide comprehensive counseling to expectant mothers and families, addressing natural history, therapeutic prognosis, genetic risks, and long-term neurodevelopmental trajectories. Post-diagnosis, serial ultrasonographic surveillance should be instituted. Fetal echocardiography monitors structural cardiac evolution, atrial septal defect dimensions, and progression of associated anomalies. Fetal d-TGA may demonstrate intrauterine disease progression; with advancing gestation, outflow tract obstruction may intensify, foramen ovale patency may become restricted or undergo premature closure, and ductal constriction may occur prematurely. Surveillance intervals of 4–6 weeks are recommended, intensifying to biweekly assessments beyond 34 weeks’ gestation, with emphasis on foramen ovale and ductal hemodynamics (Lachaud et al., 2021). Foramen ovale flow velocity exceeding 60 cm/s suggests restrictive atrial septal defect, necessitating consideration of expedited delivery or intrauterine intervention (Donofrio et al., 2014). For d-TGA fetuses with restrictive atrial septal defects, intrauterine balloon atrial septostomy has been implemented in select centers, with investigations demonstrating that for cases exhibiting foramen ovale flow velocity >100 cm/s and gestational age >32 weeks, intrauterine intervention can ameliorate postnatal hemodynamic stability while reducing urgent neonatal intervention requirements (Moon-Grady et al., 2015).

Obstetric management requires serial ultrasonographic surveillance of fetal growth parameters, amniotic fluid volume, and cervical length to promptly identify and prevent preterm delivery and fetal growth restriction. For fetal d-TGA unaccompanied by severe extracardiac malformations, pregnancy termination is not routinely recommended. Fetal TGA confers fetal distress risk comparable to normal fetuses, maintains adequate tolerance for uterine contractions, permits awaiting spontaneous labor onset, and most expectant mothers can achieve vaginal delivery; however, if intrauterine fetal distress or cardiac insufficiency manifests, or when concurrent high-risk obstetric conditions exist, planned cesarean delivery may be judiciously considered (Lachaud et al., 2021).

Peripartum individualized contingency planning should prioritize delivery during well-staffed weekday daytime hours facilitating transfer. Antenatal multidisciplinary planning is essential; delivery should occur at tertiary medical centers equipped with neonatal intensive care units (NICU), pediatric cardiac specialization, and arterial switch operation capabilities; all d-TGA neonates require postnatal oxygen saturation monitoring, with advance preparation of prostaglandin E1 and intravenous equipment, neonatal transport facilities, and coordination with referral departments for neonatal bed reservation (Donofrio et al., 2013).

Immediate postnatal echocardiographic assessment is mandatory; intravenous prostaglandin E1 maintains ductal patency. Because excessive supplemental oxygen may lower pulmonary vascular resistance and reduce ductal-level bidirectional mixing, potentially worsening relative hypoxemia, oxygen administration should be used judiciously and individualized according to oxygen saturation, acid-base status, and institutional protocols rather than applied indiscriminately; balloon atrial septostomy may be required to augment atrial-level blood mixing when indicated; prompt correction of acidosis and hypoxemia is imperative (Donofrio et al., 2013). Untreated d-TGA patients face dismal prognoses, with approximately 89% mortality within the first year; conversely, following arterial switch operation (ASO), 20-year survival exceeds 90% (Villafañe et al., 2014). Recommended surgical timing varies by d-TGA subtype: for simple d-TGA (intact ventricular septum), ASO within approximately the first week postnatally is generally favored given its association with neuroprotection, although the precise timing should be individualized based on neonatal hemodynamic stability, coronary artery anatomy, left ventricular preparedness, associated lesions, and local surgical/institutional expertise. When diagnostic delays precipitate left ventricular deconditioning (left/right ventricular pressure ratio <0.6), pulmonary artery banding for left ventricular training precedes staged ASO (Holland et al., 2015). d-TGA with VSD requires individualized timing based on VSD size, the degree of pulmonary overcirculation, and the onset of heart failure symptoms; surgery is generally pursued before significant pulmonary vascular changes or hemodynamic compromise develop. When d-TGA is complicated by VSD and significant pulmonary stenosis or left ventricular outflow tract obstruction, the arterial switch operation alone is usually not feasible; definitive repair (e.g., the Rastelli procedure) is typically deferred substantially longer—often to early childhood—to allow for adequate right ventricle-to-pulmonary artery conduit sizing and more favorable anatomy, with actual timing individualized according to coronary anatomy, the severity and anatomic substrate of the outflow obstruction, and the planned repair strategy (Alsoufi et al., 2009; Huang et al., 2019).

## 6 Pharmacological management strategies in the perinatal and perioperative period of fetal d-TGA

Intravenous suspected inadequate neurocirculatory mixing, particularly when ductal-dependent physiology, hypoxemia, or restrictive atrial-level shunting is present or anticipated; initiation should be guided by ductal patency, oxygenation, and the likely need for balloon atrial septostomy rather than applied uniformly to every neonate regardless of mixing adequacy, to preserve ductal patency and augment neurocirculatory mixing. The conventional starting dose is 0.05–0.1 g/kg/min, though accumulating evidence supports initiating therapy at substantially lower doses (0.01–0.03 g/kg/min), which achieve comparable efficacy in maintaining ductal patency while reducing dose-dependent adverse effects (Akkinapally et al., 2018). Continuous monitoring of oxygen saturation, respiratory pattern, and blood pressure is mandatory throughout infusion, with ventilatory support readily available given the risk of apnea. Dose escalation should be reserved for infants with persistent hypoxemia despite adequate ductal flow, and de-escalation to the lowest effective dose is recommended once hemodynamic stability is achieved, to minimize cumulative-dose-related toxicity (Huang et al., 2013).

Apnea, reported in approximately 10%–12% of PGE1-treated neonates, together with hyperthermia, hypotension, cutaneous flushing, and jitteriness, constitutes the most common short-term adverse effect profile. A recent randomized clinical trial demonstrated that adjunctive caffeine citrate (20 mg/kg loading dose followed by 10 mg/kg/day maintenance) significantly reduced the incidence of PGE1-induced apnea without compromising ductal patency, offering a pharmacological strategy to reduce the need for intubation solely for apnea management. Prolonged infusion (>120 h) has additionally been associated with cortical hyperostosis, gastric outlet obstruction from antral foveolar hyperplasia, and brown fat necrosis, underscoring the importance of minimizing cumulative dose and duration whenever clinically feasible (Salamati et al., 2025).

Metabolic acidosis in d-TGA neonates most commonly reflects inadequate systemic oxygen delivery secondary to restrictive intercardiac mixing rather than a primary buffering deficiency; the priority is therefore to restore effective mixing (via PGE1 and, when needed, balloon atrial septostomy) rather than reflexive sodium bicarbonate administration. Current neonatal evidence indicates that rapid or indiscriminate bicarbonate infusion may cause paradoxical intracellular acidosis, cerebral blood flow fluctuations, and osmolar load, with no demonstrated survival benefit; contemporary guidance therefore reserves bicarbonate for severe cases requiring acute correction and favors addressing the underlying cause together with balanced crystalloid volume support (Dhugga et al., 2025). Judicious volume resuscitation and correction of hypocalcemia—which can independently impair myocardial contractility and precipitate cardiogenic shock in infants, reversibly so with calcium repletion—are integral adjuncts to hemodynamic stabilization in this population (Gupta et al., 2011).

Milrinone, a phosphodiesterase-3 inhibitor with combined inotropic and pulmonary/systemic vasodilatory properties, is the most widely used perioperative agent following arterial switch operation (ASO), employed routinely in the vast majority of pediatric cardiac centers (Roeleveld and de Klerk, 2018). Classic hemodynamic studies of the early postoperative course after ASO demonstrated a characteristic fall in cardiac output 6–18 h postoperatively, often necessitating combination therapy with dopamine and/or low-dose epinephrine in addition to milrinone to support the newly “arterialized” left ventricle (Wernovsky et al., 1995). In cases of vasodilatory shock refractory to conventional catecholamines after cardiopulmonary bypass, vasopressin infusion has been used as rescue therapy (Nishibe and Tsujita, 2012).

BAS is most commonly performed at the bedside under echocardiographic guidance to minimize transport risk, and is generally conducted under general anesthesia or deep procedural sedation to ensure immobility and hemodynamic stability during catheter manipulation across the atrial septum (Weeda et al., 2024). Anesthetic choice must account for the hemodynamic fragility of cyanotic, potentially acidotic neonates; agents with minimal myocardial depressant effect and preserved sympathetic tone are generally favored, with continuous invasive or non-invasive hemodynamic monitoring throughout the procedure.

Beyond survival-oriented pharmacotherapy, several agents have been investigated for perioperative neuroprotection in infants undergoing cardiopulmonary bypass. A systematic review identified allopurinol, sodium nitroprusside, erythropoietin, ketamine, dextromethorphan, and phentolamine as candidate neuroprotective agents; notably, sodium nitroprusside was associated with lower postoperative S100β levels—a biomarker of neuronal injury—specifically in infants with transposition of the great arteries, while allopurinol reduced seizures, coma, and cardiac events in a related high-risk cohort. Dexmedetomidine has also shown promise as an intraoperative neuroprotective adjunct in pediatric cardiac surgery, though dedicated trials in d-TGA populations remain limited. These findings remain preliminary and should be framed as an emerging area rather than established practice (Stegeman et al., 2018).

## 7 Postnatal follow-up of fetal d-TGA: dual management of cardiac and neurodevelopmental outcomes

### 7.1 Long-term cardiac complication surveillance

Post-ASO management necessitates long-term monitoring for potential anatomical and functional sequelae, predominantly encompassing neo-aortic root dilatation, neo-aortic valve regurgitation, coronary artery disease, pulmonary artery stenosis, and arrhythmias. Serial echocardiography and electrocardiography constitute foundational surveillance modalities, supplemented when indicated by cardiac magnetic resonance imaging, exercise stress testing, or even coronary angiography for comprehensive assessment (Villafañe et al., 2014).

### 7.2 Long-term neurodevelopmental management

Neurodevelopmental disorders represent the most prominent challenge affecting long-term quality of life for d-TGA patients. First, aberrant fetal hemodynamics induce chronic cerebral oxygen insufficiency, manifesting as diminished total fetal brain volume, delayed cortical maturation, and abnormal cerebral metabolic profiles (Selvanathan et al., 2024; Jørgensen et al., 2018; Park et al., 2006). Second, peripartum and perioperative severe hypoxemia, acidosis, emergent interventional procedures, and cardiopulmonary bypass surgery itself confer cerebral ischemic and inflammatory risks, correlating with significantly elevated neonatal cerebral arterial resistance indices (Leon et al., 2024), thereby impairing neurodevelopment; third, chronic postoperative cardiovascular complications precipitate sustained exercise intolerance and constrained social participation (Kordopati-Zilou et al., 2022). These cumulative insults compromise neurodevelopment in fetal d-TGA, substantially elevating postnatal risks for deficits in executive function, academic achievement, social cognition, and psychobehavioral domains. Therefore, the long-term management of the neurological development of children with d-TGA is of great significance.

Jørgensen and colleagues employed longitudinal MRI to evaluate cerebral and somatic growth trajectories in d-TGA fetuses versus normal controls, revealing decelerated total brain volume growth during late gestation, with this growth retardation demonstrating progressive intensification (Jørgensen et al., 2018). Regional brain structural analyses disclosed volumetric reductions in hippocampus, corpus callosum, and basal ganglia among d-TGA fetuses compared with controls (Kelly et al., 2019). These structural alterations likely represent direct consequences of chronic fetal cerebral oxygen insufficiency and correlate closely with amplified postnatal neurodevelopmental disorder risks. Magnetic resonance spectroscopy (MRS) permits non-invasive evaluation of fetal brain metabolic states. MRS investigations of d-TGA fetuses demonstrate metabolic profile abnormalities in parietal white matter and occipital gray matter regions: diminished N-acetylaspartate (NAA)/creatine (Cr) ratios suggesting delayed neuronal maturation; elevated choline (Cho)/Cr ratios reflecting impaired myelination processes (Park et al., 2006). These metabolic alterations manifest prenatally, persist postoperatively, and inversely correlate with psychomotor developmental scores at 1 year of age (Park et al., 2006). Fetal MRI cerebral developmental measurements and metabolic assessments can serve as critical tools for d-TGA neurodevelopmental risk stratification. Selvanathan and colleagues’ 2024 prospective cohort investigation elucidated mechanistic pathways through which prenatal diagnosis confers neuroprotection for d-TGA fetuses (Selvanathan et al., 2024). This study measured neonatal brain volumes and tracked neurodevelopmental outcomes to 18 months, revealing that among postnatally diagnosed cases, smaller neonatal brain volumes significantly correlated with lower cognitive scores at 18 months; whereas in prenatally diagnosed cohorts, this association was attenuated (Selvanathan et al., 2024). Postnatal diagnosis groups exhibited significantly higher preoperative hypotension incidence compared with prenatal diagnosis groups (35% vs. 14%, P = 0.004) (Selvanathan et al., 2024), suggesting that timely prenatal diagnosis may exert neuroprotective effects by ameliorating preoperative hemodynamic status and averting severe hypotension and acidosis. For prenatally diagnosed d-TGA fetuses demonstrating pronounced cerebral developmental and metabolic profile abnormalities, comprehensive disclosure of neurodevelopmental risk to families during multidisciplinary consultations is warranted, accompanied by formulation of intensified postoperative neurodevelopmental surveillance and early intervention protocols.

Lim et al. (2019) investigation definitively established detrimental impacts of surgical delay on cerebral development. This study enrolled 45 days-TGA patients undergoing preoperative and postoperative MRI examinations, calculating brain weight Z-scores and evaluating 18-month neurodevelopment. Results demonstrated that surgical age >2 weeks associated with perioperative cerebral growth impairment and significantly correlated with diminished 18-month language developmental scores (Lim et al., 2019). Concurrent VSD and advanced surgical age constituted independent risk factors for perioperative cerebral growth impairment (Lim et al., 2019). These findings support expeditious ASO completion (within 7 days postnatally) for simple d-TGA, thereby minimally mitigating cumulative cerebral developmental damage from chronic hypoxia and pulmonary congestion. Soares et al. (2023) systematic review and meta-analysis evaluated neurodevelopmental outcomes within 5 years post-d-TGA surgery. Analysis revealed that although most patients demonstrated overall neurodevelopment within normal parameters postoperatively, deficits in specific domains including executive function, fine motor skills, and social cognition remained pervasive (Soares et al., 2023). Potentially modifiable factors encompassed preoperative hyperlactatemia and balloon atrial septostomy (Soares et al., 2023). This underscores that optimizing preoperative hemodynamic management and averting severe metabolic acidosis may favorably influence neurodevelopmental prognosis. Near-infrared spectroscopy (NIRS)-derived regional cerebral oxygen saturation (rSO_2_) monitoring has been extensively implemented in perioperative management of neonates with congenital heart disease. NIRS in neonatal intensive care monitors high-risk populations including children with congenital heart disease, providing real-time cerebral perfusion and oxygenation information conducive to individualized therapy and neuroprotection (Lin et al., 2026). Investigations targeting d-TGA arterial switch operations demonstrate that sustained preoperative, intraoperative, and postoperative cerebral oxygen desaturation correlates with adverse neurodevelopmental outcomes. Andropoulos and colleagues’ prospective research found that maintaining intraoperative rSO_2_ above 80% of baseline or absolute values > 45% significantly correlated with superior Bayley scale scores at 12 months (Andropoulos et al., 2012). Lower preoperative rSO_2_, prolonged intraoperative cardiopulmonary bypass duration, and preoperative MRI brain injury all constituted independent risk factors for declining 12-month neurodevelopmental scores (Andropoulos et al., 2012). Consequently, routine perioperative NIRS monitoring is recommended for d-TGA patients, establishing rSO_2_ thresholds (e.g., <45% or >20% decline from baseline) as intervention trigger points, maintaining adequate cerebral perfusion through anesthetic depth adjustment, hemodynamic optimization, and transfusion for anemia correction. For simple d-TGA, ASO completion within 7 days postnatally is generally recommended, when clinically feasible, to minimize prolonged hypoxic impacts on cerebral development.

Lillitos et al. (2025) report detailing 30-year follow-up data from a single UK center revealed that although cardiovascular survival rates post-ASO for d-TGA patients proved exemplary (30-day operative mortality 0.9%, long-term survival >95%), neurodevelopmental challenges remain pronounce. During school-age years and adolescence, prevalence rates for executive dysfunction, attention-deficit/hyperactivity disorder (ADHD), and autism spectrum disorders (ASD) substantially exceeded those in general populations (Lillitos et al., 2025). Approximately 65% of adolescents required special educational support, a proportion dramatically exceeding the 10%–15% observed in general populations (Szymanski et al., 2025). Long-term investigations demonstrate that even with globally normal intelligence, approximately 40%–60% of patients manifest executive dysfunction accompanied by elevated risks for attention-deficit/hyperactivity disorder, learning difficulties, and social challenges (Calderon and Bellinger, 2015). Neuroimaging likewise suggests brain structural abnormalities (including white matter microstructural alterations and hippocampal volume reductions) persist into adolescence (Bellinger et al., 2011). Hence, establishing standardized neurodevelopmental assessment protocols spanning the developmental continuum proves imperative, with targeted screening recommended at the following critical age junctures: Infancy (6, 12, 18 months): Assessing global motor and cognitive developmental milestones utilizing Bayley Scales of Infant Development (BSID) to evaluate Mental Developmental Index (MDI) and Psychomotor Developmental Index (PDI); Preschool period (3, 5 years): Emphasizing language development, emerging executive functions (inhibitory control, working memory, cognitive flexibility), fine motor skills, and social competencies; School-age period (8, 12 years): Systematically evaluating academic performance, attention, core executive functions, and social cognitive abilities; Adolescence (16 years): Transitioning toward psychosocial adaptation capacity, quality of life, and screening for anxiety, depression, and other emotional concerns (Calderon and Bellinger, 2015; Calderon et al., 2014).

Early identification coupled with systematic intervention constitutes the cornerstone for outcome amelioration. Medical institutions establishing “Cardiac Neurodevelopmental Programs” are providing early screening and therapeutic recommendations (Marino et al., 2012). These programs typically encompass: (1) standardized neurodevelopmental assessment schedules; (2) multidisciplinary teams (pediatric cardiology, neurology, developmental pediatrics, psychology, rehabilitation medicine); (3) early identification of high-risk patients with referral to early intervention services; (4) provision of educational support and resources to schools and families (Marino et al., 2012). Upon detecting developmental deviations during follow-up, individualized support should be promptly initiated, including occupational therapy, physical therapy, speech-language therapy, and targeted cognitive training. Research indicates that interventions targeting processing speed and executive function, particularly when implemented early, may facilitate academic achievement improvements while potentially mitigating stress and self-confidence erosion associated with academic underperformance (Cassidy et al., 2016). Concurrently, providing families with sustained psychological support and educational guidance helps them comprehend and address their children’s potential “invisible” cognitive challenges, collectively promoting optimal long-term adaptation for affected patients.

## 8 Conclusions and future perspectives

**FIGURE 1:**
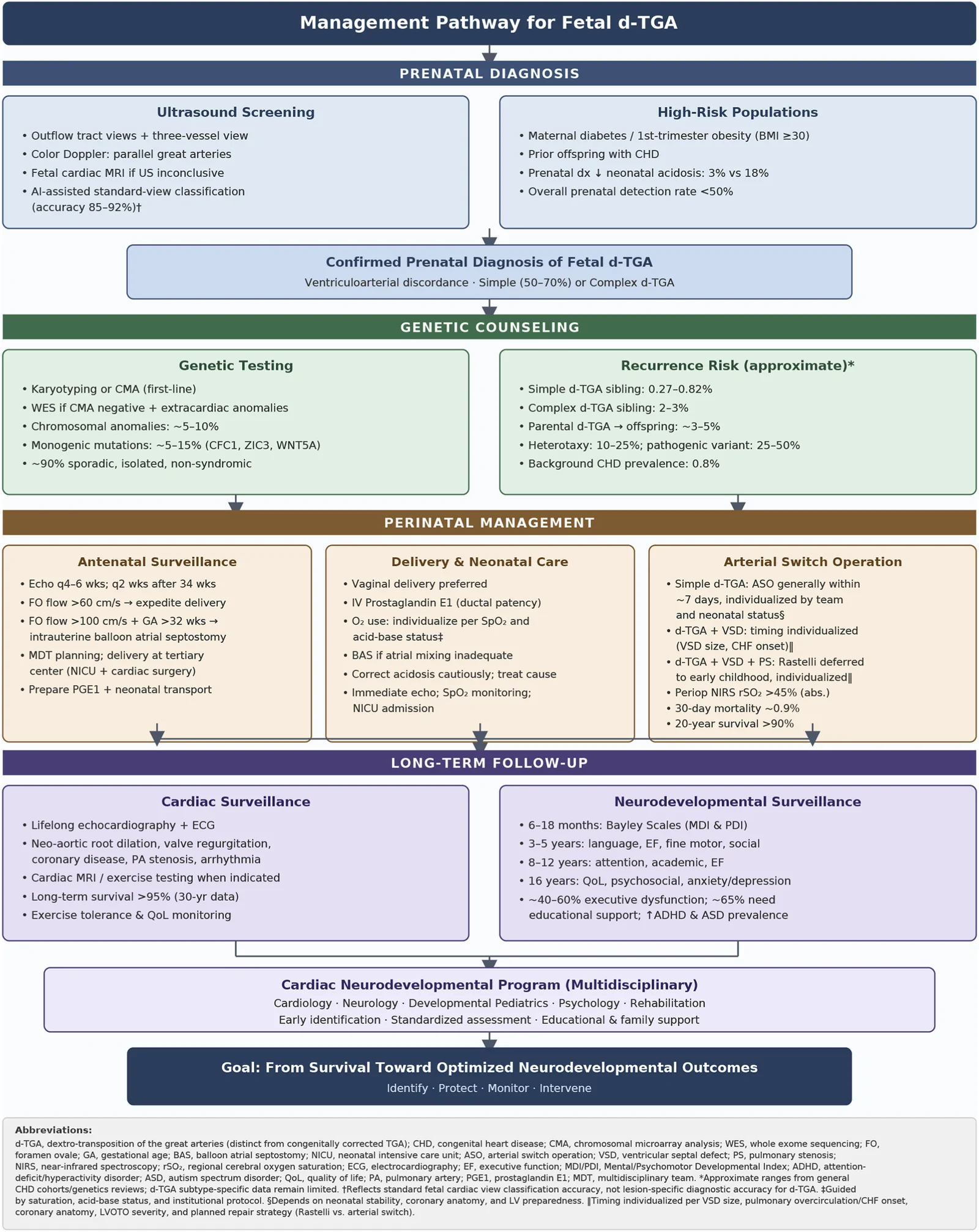
The management pathway of fetal d-TGA.

In summary, clinical management of fetal d-TGA has successfully transcended the survival barrier, yet this very achievement has illuminated a long-obscured challenge: approximately half of affected children experience neurodevelopmental complications including executive dysfunction, learning difficulties, and social adaptation challenges that profoundly compromise their quality of life. This review elucidates the intricate interplay between environmental and genetic factors in fetal d-TGA pathogenesis, showcases the pivotal roles of AI-assisted technologies and fetal MRI in enhancing prenatal detection rates and cerebral developmental risk stratification, establishes evidence-based recurrence risk assessment criteria for different d-TGA subtypes to inform genetic counseling, constructs an integrated management pathway encompassing intrauterine surveillance, multidisciplinary collaboration, perinatal care, and neonatal resuscitation, and proposes a comprehensive neurodevelopmental management strategy spanning prenatal risk identification, perioperative neuroprotection, and long-term postoperative follow-up (Figure 1). These advances collectively converge upon a fundamental transformation: clinical management of d-TGA must evolve from “saving lives” to “optimizing long-term outcomes.” Future investigations should concentrate on deeper mechanistic insights, establishment of risk prediction models, refinement of neuroprotective strategies, and evidence-based validation of early intervention protocols, constructing a holistic “identify-protect-monitor-intervene” management paradigm to genuinely achieve the ultimate goal of transitioning d-TGA patients from survival toward optimized long-term neurodevelopmental outcomes.
